# *Lithospermum erythrorhizon* extract alleviates immunosuppression via MAPK signaling pathway

**DOI:** 10.3389/fvets.2025.1654212

**Published:** 2025-09-29

**Authors:** Xiaoqing Chi, Li Ping, Renhua Gai, Qinjie Weng

**Affiliations:** College of Pharmaceutical Sciences, Zhejiang University, Hangzhou, Zhejiang, China

**Keywords:** *Lithospermum erythrorhizon* extract, immunosuppression, inflammation, MAPK signaling, network pharmacology

## Abstract

**Introduction:**

*Lithospermum erythrorhizon* extract (LEE), rich in shikonin and its derivatives, has been traditionally valued for anti-inflammatory and wound-healing properties.

**Objective:**

This study aimed to investigate the immunoprotective effects and underlying mechanisms of LEE in a rat model of dexamethasone-induced immunosuppression.

**Methods:**

One hundred SPF Sprague–Dawley rats were randomized into control, model, and LEE treatment groups (10, 20, 40 mg/kg). Immunosuppression was induced with dexamethasone (7.5 mg/kg, i.p.) for 7 days, followed by oral LEE for 21 days. Body weight, food consumption, hematology, and serum biochemistry were assessed. Immunomodulatory effects were evaluated via cytokine profiles, immunoglobulin and complement levels, lymphocyte subtypes and proliferation, immune organ indices, and histopathology. Potential targets and pathways were predicted by network pharmacology and validated by RT-qPCR.

**Results:**

LEE significantly improved body weight, white blood cell counts (WBC), lymphocyte (LYMPH%), and CD4^+^/CD8^+^ ratio. It downregulated pro-inflammatory cytokines (tumor necrosis factor-*α*, interleukin-1β, interleukin-6) (TNF-α, IL-1β, IL-6) and upregulated anti-inflammatory cytokine interleukin-4 (IL-4), while restoring immunoglobulin G, M and A (IgG, IgM, IgA) and complement 3 and 4 (C3, C4) levels. LEE also enhanced ConA- and LPS-induced lymphocyte proliferation, and alleviated spleen and thymus atrophy, as evidenced by increased organ indices and improved histopathology. Network pharmacology highlighted MAPK signaling, particularly the p38 and JNK- as central pathways, which was supported by RT-qPCR showing upregulation of Akt1, Mapk3, Mapk14, Pik3ca, and Mapk1.

**Conclusion:**

LEE effectively ameliorates dexamethasone-induced immunosuppression by restoring immune cell activity, regulating cytokine balance, and preserving immune organ structure, primarily via MAPK pathway regulation. This study provides a scientific basis for the development of LEE as a natural immunomodulatory agent in managing immunosuppression in mammals.

## 1 Introduction

Immunosuppression, characterized by a compromised immune system that diminishes its ability to defend against pathogens and malignancies, represents a formidable challenge in healthcare. This condition can result from diverse causes, including pharmacological interventions, chronic diseases, and specific medical procedures. The impaired immune response significantly increases susceptibility to infections, delays wound healing, and elevates the risk of cancer progression and transplant rejection, all of which complicate clinical outcomes and hinder recovery (1–3). Notably, one critical yet often underappreciated factor contributing to immunosuppression is chronic inflammation, which disrupts immune homeostasis by inducing cytokine imbalances and promoting immune cell exhaustion (4). Efforts to mitigate immunosuppression require a delicate balance—refining therapeutic approaches to preserve immune functionality while minimizing adverse effects. Central to this endeavor is understanding the interplay between immunosuppression and inflammation, as chronic inflammatory responses can exacerbate immune dysfunction and drive disease progression. This intersection highlights the pressing need for innovative strategies, including the exploration of natural products with immunomodulatory and anti-inflammatory potential, to restore immune homeostasis and enhance therapeutic outcomes.

Inflammation, a pivotal component of the body’s innate defense system, serves as a double-edged sword in immunological balance. While acute inflammation is essential for combating infections and initiating tissue repair, chronic or dysregulated inflammation can paradoxically drive immunosuppression. Chronic inflammation disrupts cytokine homeostasis, as evidenced by elevated levels of pro-inflammatory mediators such as tumor necrosis factor-*α* (TNF-α) and interleukin-1β (IL-1β), which not only amplify the inflammatory cascade but also suppress T cell activity and impair immune coordination (5, 6). Moreover, chronic inflammatory states are associated with diminished immunoglobulin synthesis, resulting in reduced antibody production and a compromised humoral immune response. Pathological remodeling of primary lymphoid organs, including the spleen and thymus, further exacerbates immune dysfunction by impairing the maturation and functionality of immune cells (7). Breaking this inflammatory-immunosuppressive loop remains a critical goal in the development of next-generation immunotherapeutics. At the molecular level, chronic inflammation is closely linked to the dysregulation of key signaling pathways, particularly the nuclear factor κB (NF-κB) and mitogen-activated protein kinase (MAPK) pathways. Persistent activation of the NF-κB pathway leads to sustained expression of pro-inflammatory cytokines, chemokines, and adhesion molecules, thereby maintaining a chronic inflammatory state that exhausts immune resources (8). Simultaneously, activation of the MAPK signaling cascade—especially through the p38 (MAPK) and JNK (c-Jun N-terminal Kinase) branches—contributes to the upregulation of IL-1β and TNF-*α*, which in turn suppress T-cell differentiation and inhibit antigen-presenting functions (9). These molecular events serve as critical mediators connecting unresolved inflammation to functional immunosuppression. This intricate interplay between inflammation and immunosuppression underscores the critical need for interventions capable of breaking this vicious cycle by modulating the signaling networks at the crossroads of immune activation and suppression.

**Figure 1:**
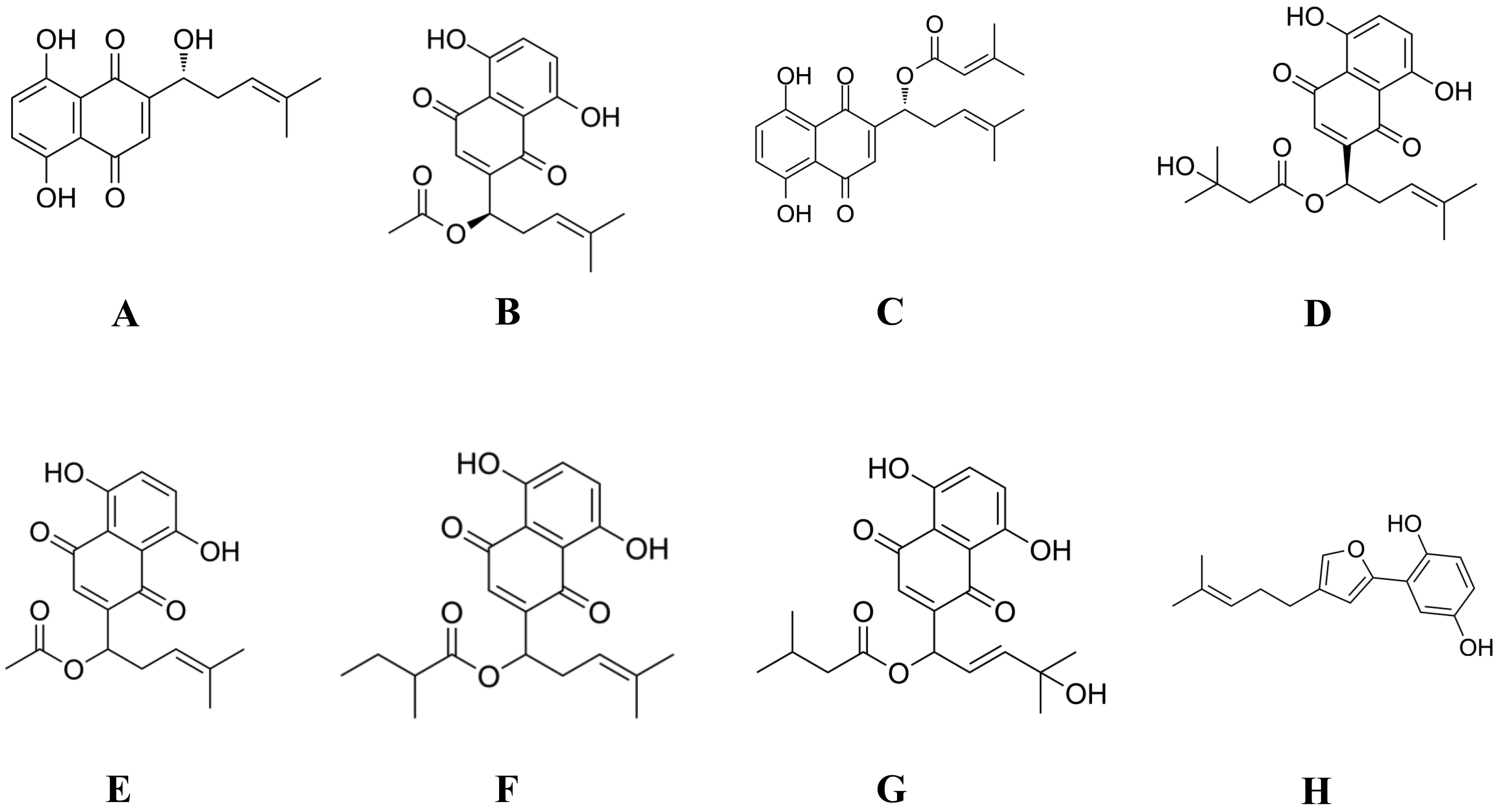
Chemical structures of the major compounds identified in LEE and their derivatives. **(A)** Shikonin; **(B)** Acetylshikonin; **(C)** β,β-Dimethylacrylshikonin; **(D)** β-Hydroxyisovalerylshikonin; **(E)** DL-Acetylshikonin; **(F)** (2-Methylbutyryl) shikonin; **(G)** Lithospermidin B; **(H)** Deoxyshikonofuran.

Natural medicines have long been recognized for their potential to enhance anti-inflammation and immune enhancement function. Among these, *Lithospermum erythrorhizon*, a traditional Chinese medicinal herb rich in bioactive compounds such as shikonin, β,β-dimethylacrylshikonin and other naphthoquinones, has long been recognized for its anti-inflammatory effects. Research has shown that shikonin effectively suppresses pro-inflammatory cytokines, thereby reducing chronic inflammation and alleviating related conditions (10). Chemically, over 80 distinct compounds have been identified from *Lithospermum erythrorhizon*, primarily includes shikonin, acetylshikonin, isobutyrylshikonin, and β,β-dimethylacrylshikonin, which are responsible for the characteristic red pigment of the root (Figure 1) (11). These compounds have shown significant potential in modulating immune responses and exerting anti-inflammatory effects. Among them, shikonin has been widely studied for its immunomodulatory activity, including its ability to enhance macrophage phagocytosis, stimulate dendritic cell maturation, and promote antigen presentation (12). Acetylshikonin has demonstrated the ability to inhibit pro-inflammatory cytokine production, thus aiding in the regulation of immune homeostasis and the prevention of excessive immune activation (13). Furthermore, Li et al. indicates that shikonin derivatives can influence T-cell differentiation and boost the activity of natural killer (NK) cells, contributing to the body’s defense against pathogens and tumors (14). In addition, some studies have shown that shikonin and its analogs can modulate the expression of key cytokines such as interleukin-2 (IL-2), interferon-*γ* (IFN-γ), and TNF-*α*, which are essential for orchestrating immune responses (15, 16). These findings highlight the potential of *Lithospermum erythrorhizon* extract (LEE) as a natural source of immunoregulatory agents, offering promising prospects for enhancing immune health and developing novel therapeutic strategies. By exploring the therapeutic synergy of natural compounds, we aim to uncover innovative strategies to address the intertwined challenges of inflammation and immune dysregulation. Despite growing interest in natural immunomodulators, the molecular targets and pathways through which LEE exerts its immunoprotective effects remain insufficiently elucidated. To bridge this gap, network pharmacology has emerged as a powerful systems-level approach that integrates compound-target prediction, protein–protein interaction (PPI) analysis, and pathway enrichment, allowing the identification of key regulatory networks involved in disease modulation. In this study, network pharmacology was applied to systematically screen and analyze the potential targets of LEE in the context of immunosuppression. Among the core targets identified were protein kinase Bα (Akt1), mitogen-activated protein kinase 3 (Mapk3), mitogen-activated protein kinase 1 (Mapk1), mitogen-activated protein kinase 14 (Mapk14), and Pik3ca, which are central components of the MAPK and PI3K-Akt signaling pathways—both of which are critical for immune cell survival, differentiation, and inflammatory regulation.

Therefore, this study aims to comprehensively investigate the immunoprotective effects of LEE in a rat model of dexamethasone-induced immunosuppression, by integrating traditional pharmacodynamic evaluation with network pharmacology and gene expression validation, thus providing mechanistic insight into its multi-target immunomodulatory actions and supporting its potential clinical application as a natural immunopotentiator.

## 2 Materials and methods

### 2.1 Animals

SPF SD rats, weighing 80 ~ 100 g, were purchased from Vitalriver Co., Ltd., (animal license number SCXK 2021–0006). The rats were housed at the Center for Drug Safety Evaluation and Research at Zhejiang University, under controlled environmental conditions: temperature maintained at 20 ~ 26 °C, relative humidity at 40 ~ 70%, and a 12-h light cycle (from 8:00 to 20:00). They were provided ad libitum access to food and water and underwent a 7-day acclimatization period. All animal procedures were conducted in accordance with the guidelines approved by the Institutional Animal Care and Use Committee of the Center for Drug Safety Evaluation and Research, Zhejiang University (IACUC No. 23-s087).

### 2.2 Chemicals

*Lithospermum erythrorhizon* root was purchased from Shaanxi Honghao Bio-Tech Co., Ltd. (Shanxi, China). Shikonin (565850), β,β-Dimethylacrylshikonin (SML3463), acetylshikonin (TA9H93CFC329), pentobarbital sodium (20121030) and 3-(4, 5-dimethyl-2-thiazolyl)-2, 5-diphenyl-2-H-tetrazolium bromide (MTT, M2128) were purchased from Sigma-Aldrich Inc. (St. Louis, MO, United States). Isobutyrylshikonin (B11182) was purchased from Shanghai Shifeng Bio-Technology Co., Ltd. Anhydrous ethanol, methanol, acetonitrile, cyclohexane, petroleum ether, ethyl acetate, and acetone were purchased from Sinopharm Chemical Reagent Co., Ltd. (Beijing, China). Sodium chloride injection was purchased from Hangzhou Minsheng Pharmaceutical (Hangzhou, China). Phosphate-buffered saline (PBS) (10010023), RPMI-1640 medium (7200047), TNF-*α* (400-14) and interleukin-1β (IL-1β, 400-01B) ELISA kits were the products of Gibco (Thermo Fisher Scientific, Waltham, United States). Interleukin-4 (IL-4, SEKR-0004), interleukin-6 (IL-6, SEKR-0005), immunoglobulin A (IgA, SEKR-0018), immunoglobulin G (IgG, SEKR-0020), immunoglobulin M (IgM, SEKR-0021) ELISA kits were the products of Solarbio Co. Ltd. (Beijing, China). Anti-CD4 antibody (OX-35) and Anti-CD8 antibody (341) were the products of Abcam (Cambridge, United Kingdom). Alanine aminotransferase (ALT, 336794), aspartate aminotransferase (AST, 348556), albumin (Alb, 322,975), creatinine (Cr, 340,775), glucose (Glu, 330,126), total cholesterol (TC, 347559), total protein (TP, 335972), urea nitrogen (BUN, 354868) and triglyceride (TG, 241813) were purchased from Roche Diagnostics GmbH (North America, United States). Complement 3 (C3, CSB-E08666r) and Complement 4 (C4, CSB-E08706r) ELISA kits were the products of Cusabio Technology LLC. (Houston, United States). RNAiso™ Plus kit (9108) and PrimeScript™ RT reagent kit (6210A) were the products of Takara (Dalian, China). Diluent PK-30 (G7444), Leukolysin FFD-200A (R7078), Dye solution FS-800A (A7114), Basophilic hemolysin FBA-200A (R7070) and Hemolysin SLS-220A (A7016) were purchased from Jinan Xisen Meikang Medical Electronics Co., Ltd (Jinan, China).

### 2.3 Instruments

High performance liquid chromatography (HPLC, LC-20A, Shimadzu); Electronic balance (PL2001-L, Mettler Toledo); Electronic Analysis Balance (MS105DU, Mettler Toledo); Electronic Analysis Balance (AL104, Mettler Toledo); Clean Bench (JB-VD-650 U, Suzhou Jiabao Purification Engineering Equipment); pH Meter (PB-21, Sartorius); Water Purification Equipment (Purelab OptionS7, ELAG); Hematology analyzer (XT-2000i, Sysmex); Serum chemistry analyzer (Cobas C311, Roche); Flow cytometer (FACSCalibur) (BD Biosciences, United States); Microplate reader (Multiskan FC, Thermo Fisher Scientific); PCR (CFX96 Touch, Bio-Rad); RNA/DNA quantification analyzer (NanoDrop^®^ ND-1000, Thermo Fisher Scientific); Microscope (DM4000, Leica); Biological Microscope (E5, Ningbo Shunyu).

### 2.4 Methods

#### 2.4.1 LEE preparation

The *Lithospermum erythrorhizon* extract (LEE) was prepared by a combination of optimized ultrasonic-assisted extraction and high-pressure extraction methods, with slight modifications based on the protocol reported by Kim et al. (17). Briefly, dried roots of *L. erythrorhizon* were ground into coarse powder and extracted using 70% ethanol at a solid-to-liquid ratio of 1:20 (w/v). The extraction process involved ultrasonic treatment at 40 kHz for 30 min followed by high-pressure extraction at 100 bar for 20 min. The combined extract was filtered, concentrated under reduced pressure, and then lyophilized to obtain a dry powder. The yield of the final extract was approximately 43.26% (w/w) based on the dry weight of raw materials. HPLC analysis indicated that the extract contained 5.62% (w/w) of shikonin and its derivatives as the major active components.

#### 2.4.2 Experimental design

**Figure 2:**
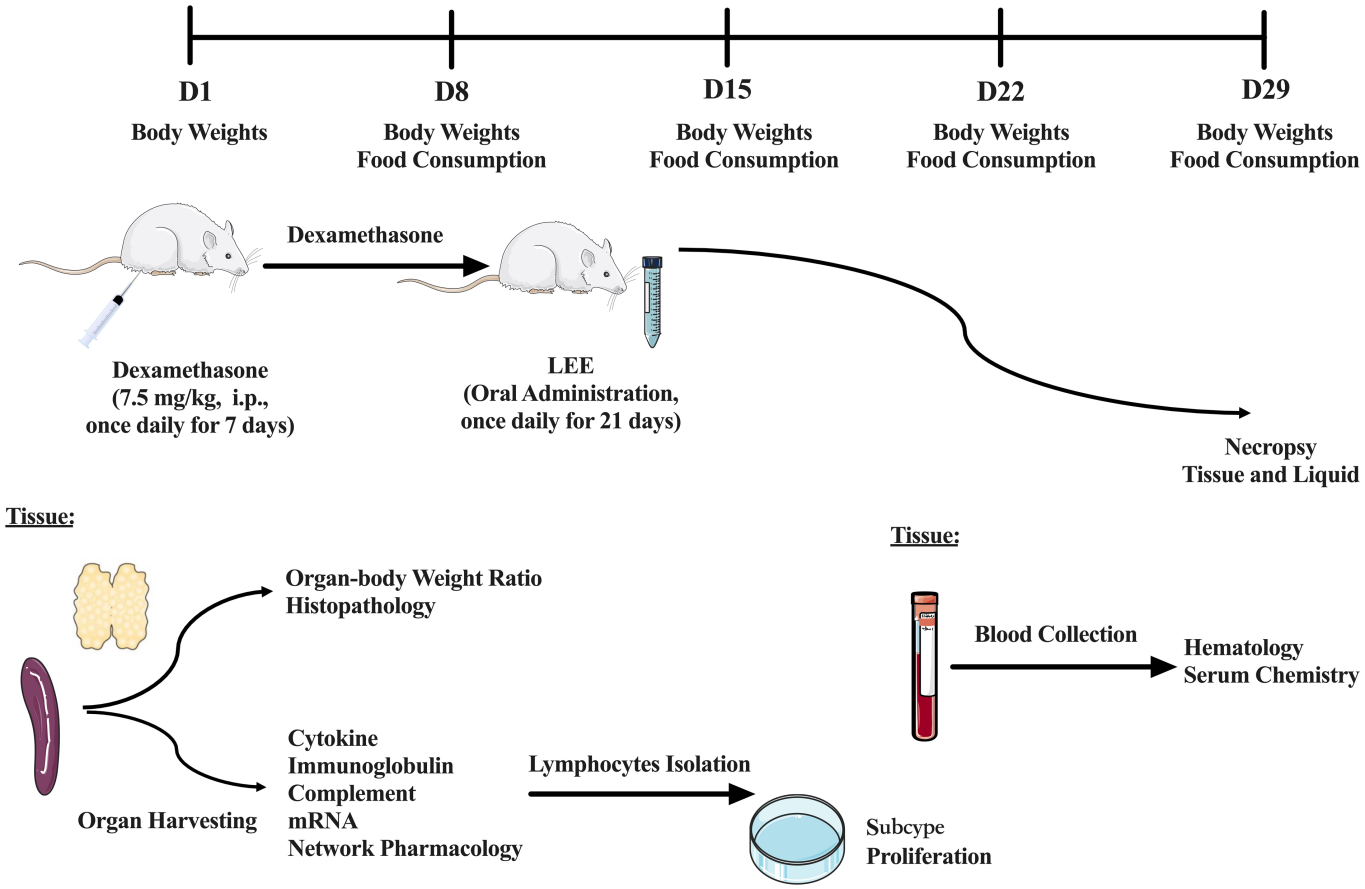
Schematic diagram of the experimental design and timeline. A total of 100 rats were randomly assigned into five groups (*n* = 20 per group): control, model, low dose (10 mg/kg), medium dose (20 mg/kg), and high dose (40 mg/kg) of LEE. Immunosuppression was induced via daily intraperitoneal injection of dexamethasone (7.5 mg/kg) for 7 consecutive days (Days 1–7). LEE was administered orally once daily for 21 days (Days 8–28). Body weight and food consumption were monitored weekly. At the end of the treatment period, blood samples were collected for hematology and serum chemistry analysis. Additionally, spleen was harvested for the evaluation of cytokine, immunoglobulin, complement level and mRNA expression. Lymphocytes were isolated and analyzed for subtype classification and proliferation capacity. Organ-body weight ratios and histopathology examinations were also performed. To further elucidate the potential mechanisms of LEE, network pharmacology analysis was conducted to predict candidate targets, which were subsequently validated by RT-qPCR in spleen tissues.

A stratified randomization approach was used to allocate 100 selected animals into five groups (Table 1): control, model, low dose (10 mg/kg), medium dose (20 mg/kg), and high dose (40 mg/kg), with 20 rats per group. The dosages of LEE were determined based on preliminary dose-ranging studies (data not shown), while immunosuppression was induced by intraperitoneal injection of the dexamethasone (7.5 mg/kg, i.p., once daily for 7 consecutive days), according to previously research (18). Animals in the dose groups received daily oral administration of LEE for 21 consecutive days following dexamethasone treatment, the first day of dexamethasone administration was designated as Day 1. Body weight and food consumption were recorded weekly. At the end of the LEE administration period, blood samples were collected for hematology and serum chemistry analysis. Additionally, spleen (*n* = 10) was harvested for the evaluation of cytokine, immunoglobulin, complement level and messenger ribonucleic acid (mRNA) expression. Lymphocytes were isolated and analyzed for subtype classification and proliferation capacity. Organ-body weight ratios and histopathology examinations (*n* = 10) were also performed. To further elucidate the potential mechanisms of LEE, network pharmacology analysis was conducted to predict candidate targets, which were subsequently validated by real-time quantitative PCR (RT-qPCR) in spleen tissues. Details of the experimental grouping and study design are provided in Table 1 and Figure 2.

**Table 1:** Grouping.

SN	Group	N	Dexamethasone (mg/kg)	LEE (mg/kg)
1	Control	20	0	0
2	Model	20	7.5	0
3	Low dose	20	7.5	10
4	Medium dose	20	7.5	20
5	High dose	20	7.5	40

#### 2.4.3 Body weights

Animals were weighed prior to the first dexamethasone administration, and subsequently on Day 8, 15, 22, and 29 of the dosing periods.

#### 2.4.4 Food consumption

Food consumption was measured weekly by subtracting the remaining feed from the total feed added over 7-day intervals. Average daily food consumption per rat was calculated using the following formula:


$$\mathrm{Food Consumption}=\frac{\left(\mathrm{Food Addition}-\mathrm{Food Remaining}\right)∕\mathrm{Days}}{\mathrm{Animal Numbers}\;\mathrm{per}\;\mathrm{Cage}}$$

#### 2.4.5 Hematology examination

At the end of the administration period, blood samples were collected from the anesthetized rats via the abdominal aorta using vacuum blood collection needles. The samples were immediately transferred into EDTA dipotassium salt (EDTA-K2) anticoagulant tubes. Hematology parameters were analyzed as outlined in Supplementary Table 1 (19).

#### 2.4.6 Serum chemistry examination

Following the final dosing, blood was collected from the abdominal aorta of anesthetized rats using vacuum blood collection needles and transferred into tubes containing coagulation accelerators. After clotting and centrifugation, serum chemistry parameters were measured as detailed in Supplementary Table 2 (20).

#### 2.4.7 Cytokine measurement

On day 29 (following the final administration), the spleen homogenates were prepared and centrifuged to collect the supernatant (1:5 dilution) for cytokine analysis. Levels of TNF-*α*, IL-1β, IL-4, and IL-6 were quantified using ELISA kits and expressed in pg./mL, following the manufacturer’s instructions (21).

#### 2.4.8 Immunoglobulin and complement measurement

On day 29, the spleen was harvested to access the immunoglobulin and complement levels. IgG, IgM, IgA, C3, and C4 levels were determined using ELISA kits according to the manufacturer’s instructions (22).

#### 2.4.9 Real-time quantitative PCR expression

Total RNA was extracted from spleen tissues using the RNAiso™ Plus kit according to the manufacturer’s instructions. RNA purity and integrity were confirmed by evaluating the 260/280 nm absorbance ratio (ranging from 1.8 to 2.0), and RNA concentration was determined using a NanoPhotometer spectrophotometer. cDNA synthesis was then performed using the PrimeScript™ RT reagent kit, following the manufacturer’s protocol. Primers used for RT-qPCR were designed with Primer 5 software and synthesized by Takara (Takara, Dalian, China) and are listed in Supplementary Table 3. β-Actin was used as the internal control to normalize gene expression. The mRNA expression levels of Interferon-*γ* (IFN-γ), IL-6, IL-1β, vascular cell adhesion molecule-1 (VCAM-1), intercellular adhesion molecule 1 (ICAM-1) and NF-κB were detected by performing RT-qPCR reactions on Bio-Rad T100 (Bio-Rad Laboratories, Inc., United States) (23). The comparative Ct value method was used to quantify mRNA expression relative to β-actin expression using the 2^−ΔΔ^CT method, and results were expressed as mean ± standard deviation (SD).

#### 2.4.10 Lymphocyte subtype analysis

On day 29 (after the administration period), spleens were collected and processed for cell suspensions. Cell viability was assessed via trypan blue exclusion, and the cell suspension was adjusted to a concentration of 5.0 × 10^6^ cells/mL. For flow cytometry analysis, cells were stained with CD4-FITC and CD8-PE antibodies, incubated in the dark at room temperature for 30 min, centrifuged at 1,200 g for 5 min, and resuspended in 0.3 mL PBS. The CD4^+^, CD8^+^, and CD4^+^/CD8^+^ ratios were analyzed using FlowJo V10 software (24).

#### 2.4.11 Lymphocyte proliferation assay

Lymphocytes were prepared as described in section 2.4.10. Proliferative capacity was assessed using the MTT assay (25). Briefly, cells were stimulated with Concanavalin A (ConA, 5 μg/mL) and Lipopolysaccharide (LPS, 8 μg/mL), and optical density (OD) was measured at 570 nm after the addition of MTT and solubilization in DMSO (with 0.04 N HCl). The stimulation index (SI) was calculated as follows: SI = OD value of mitogen-stimulated cells (ConA-5 μg/mL, LPS-8 μg/mL) divided by OD value of non- mitogen-stimulated cells.

#### 2.4.12 Organ weights

During necropsy, the absolute weights of the spleen and thymus were measured. The “organ-body weight ratio” was calculated using the following formula.


$$\mathrm{Organ}-\mathrm{Body Weight Ratio}=\frac{\mathrm{Absolute Weight of Organ}\;\left(g\right)}{\mathrm{Body Weights}\;\left(\mathrm{kg}\right)\times 1000}\times 100\%$$

#### 2.4.13 Histopathology examination

Spleens and thymuses from all groups were fixed in buffered formalin, sectioned, and stained with hematoxylin and eosin (HE method) for histopathology evaluation.

#### 2.4.14 Computational system pharmacology analysis

##### 2.4.14.1 Identification and screening of active components

Active compounds of LEE were initially identified through the Traditional Chinese Medicine Systems Pharmacology (TCMSP) database,^1^ using oral bioavailability (OB ≥ 30%) and drug-likeness (DL ≥ 0.18) as screening criteria. To ensure relevance to the test extract, these compounds were further cross-validated with the main constituents identified by HPLC analysis performed in the current study. This dual-approach ensured both database-driven and experimentally-confirmed reliability of selected bioactive molecules (26).

##### 2.4.14.2 Target prediction

The molecular targets of the screened active compounds were predicted using SwissTargetPrediction^2^ (based on chemical similarity and known bioactivity information in humans) and Similarity Ensemble Approach (SEA)^3^ (which uses ligand-based chemical similarity to infer biological targets). Predicted targets were standardized using UniProt database annotations^4^ and limited to *Homo sapiens* proteins for consistency (26).

##### 2.4.14.3 Screening of potential compound targets associated with immunosuppression

To determine the potential therapeutic relevance of the predicted targets, immunosuppression-related genes were collected from several publicly available databases, including GeneCards, OMIM, and DisGeNET, using “immunosuppression” and related keywords. Compound-related targets were intersected with disease-related genes to identify common targets using Venny 2.1.0.^5^ The overlapping genes were then imported into Cytoscape (v3.9.1) to construct a compound–target–disease network. Network topology was analyzed to identify key nodes with high degree values representing potential core targets (26).

##### 2.4.14.4 Protein–protein interaction network analysis

The intersected targets were submitted to the STRING database (v11.5)^6^ to construct a protein–protein interaction (PPI) network with a minimum required interaction score of 0.7 (high confidence). The resulting PPI network was imported into Cytoscape, and topological analysis was performed using the CytoHubba plugin to determine core targets based on multiple centrality parameters, including degree, betweenness centrality, and closeness centrality. The top-ranked hub genes were selected for further functional and experimental validation (26).

##### 2.4.14.5 Gene ontology and pathway analysis

To explore the biological significance of the overlapping targets, Gene Ontology (GO) functional enrichment (covering biological processes, molecular functions, and cellular components) and Kyoto Encyclopedia of Genes and Genomes (KEGG) pathway enrichment analyses were conducted using the Metascape platform.^7^ Enrichment terms with *p* < 0.05 and enrichment factors > 1.5 were considered statistically significant. The results were visualized using bar plots and bubble charts to highlight key biological processes and signaling pathways, particularly those involved in immune regulation, inflammation, and cellular signaling cascades (26).

#### 2.4.15 Validation of mechanism by RT-qPCR

To experimentally validate the core regulatory targets identified from the network pharmacology analysis, RT-qPCR was performed. Total RNA was extracted from spleen tissues using the RNAiso™ Plus kit, and cDNA synthesis was conducted using the PrimeScript™ RT reagent kit according to the manufacturer’s protocol, as described in Section 2.4.9. The mRNA expression levels of Akt1, Mapk3, Mapk1, Mapk14, and Pik3ca—representing key nodes in the MAPK and PI3K-Akt signaling pathways—were measured and normalized to the housekeeping gene GAPDH. The relative expression levels were calculated using the 2^-ΔΔ^CT method. All reactions were performed in triplicate. Data were expressed as mean ± standard deviation (SD) from at least three independent biological replicates. The primer sequences used in this study are listed in Supplementary Table 3 (27).

#### 2.4.16 Statistics and analysis

Statistical analysis was performed using SPSS Statistics for Mac (Version 20, IBM, United States) with one-way analysis of variance (ANOVA), followed by Tukey’s *post-hoc* test for multiple comparisons between groups. All tests were two-sided, and statistical significance was defined as *p* < 0.05, *p* < 0.01 or *p* < 0.001. Results were expressed as means ± standard deviation (SD). GraphPad InStat software (Version 7, GraphPad, United States) was used to assist with statistical visualization.

## 3 Results

### 3.1 LEE preparation

The optimized extraction procedure involved three successive rounds of reflux extraction with 70% ethanol (v/v), each lasting 2 h, using a solid-to-liquid ratio of 1:20 (w/v). After pooling the extracts, the combined solution was filtered and concentrated under reduced pressure, followed by vacuum drying to obtain a purple-red powder. The final extract yield was calculated based on the dry weight of the raw material. Quantitative analysis using validated HPLC methods revealed that the major active constituents in the extract were shikonin (3.28%, w/w), acetylshikonin (1.09%, w/w), isobutyrylshikonin (0.37%, w/w), and β,β-dimethylacrylshikonin (0.88%, w/w), as summarized in Table 2. These naphthoquinone derivatives are known for their immunomodulatory and anti-inflammatory properties and serve as key pharmacologically active markers of *Lithospermum erythrorhizon*.

**Table 2:** HPLC analysis of active ingredients in LEE (paste 5 mg/mL).

Items	Percentage (%)
Shikonin	3.28
Acetylshikonin	1.09
β,β-Dimethylacrylshikonin	0.88
Isobutyrylshikonin	0.37

### 3.2 Body weights

There were no significant differences in body weight among the groups prior to modeling. By Day 8 (after 1 week of administration), the model group exhibited a significant reduction in body weight compared to the control group (*p* < 0.01), whereas the medium and high dose groups showed significantly higher body weights than the model group (*p* < 0.05). On Day 15, body weights in the medium and high dose groups remained significantly higher than those in the model group (*p* < 0.05). By Day 22, all treatment groups exhibited significantly increased body weights compared to the model group (*p* < 0.01), with no significant differences observed between the medium or high dose groups and the control group (*p* > 0.05). On Day 29, the medium and high dose groups continued to show significantly higher body weights than the model group (*p* < 0.001), and their weights were comparable to those of the control group. These results suggest that LEE effectively mitigated dexamethasone-induced weight loss (Supplementary Table 4).

### 3.3 Food consumption

No significant differences in food consumption were observed among the treatment groups compared to the control group throughout the study period. Detailed results are presented in Supplementary Table 5.

### 3.4 Hematology examination

Compared with the control group, the model group exhibited a significant reduction in white blood cell (WBC) count (*p* < 0.001). Both the medium and high dose treatment groups showed significantly increased WBC counts compared to the model group (*p* < 0.001), with the high dose group nearly restoring WBC levels to those of the control group. In addition, dexamethasone administration significantly reduced the percentage of lymphocytes (LYMPH%) (*p* < 0.001). After treatment with LEE, LYMPH% significantly increased (*p* < 0.001), showing no significant difference from the control group (*p* > 0.05). These findings are summarized in Table 3.

**Table 3:** Hematology examination (mean ± SD, *n* = 20).

SN	Group	Parameters				
		WBC (10^9^/L)	NEUT% (%)	LYMPH% (%)	MONO% (%)	EO% (%)
1	Control	9.1 ± 1.5^a^	12.3 ± 2.8	88.2 ± 3.3^a^	2.2 ± 0.8	0.6 ± 0.2
2	Model	5.9 ± 1.9^c^	9.4 ± 3.8	67.4 ± 4.7^b^	3.0 ± 0.3	0.7 ± 0.4
3	Low dose	6.9 ± 1.0^bc^	10.1 ± 3.2	84.2 ± 3.4^ab^	2.6 ± 0.6	0.7 ± 0.3
4	Medium dose	8.0 ± 1.7^b^	11.4 ± 2.4	85.2 ± 3.0^ab^	2.9 ± 1.0	0.5 ± 0.2
5	High dose	8.7 ± 1.4^ab^	12.3 ± 3.2	84.2 ± 3.1^ab^	2.7 ± 0.7	0.7 ± 0.3
		**BASO% (%)**	**RBC (10^12^/L)**	**Hb (g/dL)**	**Hct (%)**	**MCV (fL)**
1	Control	0.0 ± 0.0	7.3 ± 0.2	14.3 ± 0.6	40.8 ± 1.2	57.6 ± 1.0
2	Model	0.0 ± 0.0	6.7 ± 0.3	13.8 ± 0.5	40.9 ± 1.1	56.3 ± 1.4
3	Low dose	0.0 ± 0.0	6.9 ± 0.5	14.0 ± 0.4	41.0 ± 1.7	58.8 ± 2.6
4	Medium dose	0.0 ± 0.0	7.1 ± 0.2	14.1 ± 0.5	40.1 ± 1.5	57.1 ± 1.3
5	High dose	0.0 ± 0.0	6.8 ± 0.4	13.7 ± 0.5	41.4 ± 1.4	59.4 ± 2.3
		**MCH (pg)**	**MCHC (g/dL)**	**PLT (10^9^/L)**		
1	Control	20.9 ± 0.3	34.7 ± 0.6	1,017 ± 125		
2	Model	18.7 ± 0.6	29.3 ± 0.5	877 ± 280		
3	Low dose	20.5 ± 1.0	34.0 ± 0.2	933 ± 241		
4	Medium dose	19.1 ± 0.4	34.4 ± 0.3	1,033 ± 188		
5	High dose	20.0 ± 0.4	33.8 ± 0.7	961 ± 172		

Different letters are significantly different (*P* < 0.05, *P* < 0.01, or *P* < 0.001).

### 3.5 Serum chemistry examination

No significant differences were observed in serum chemistry parameters among the experimental groups compared to the control group. Detailed data are presented in Table 4.

**Table 4:** Serum chemistry examination (mean ± SD, *n* = 20).

SN	Group	Parameters				
		TP (g/L)	Alb (g/L)	ALT (U/L)	AST (U/L)	BUN (mmol/L)
1	Control	55.7 ± 2.5	38.5 ± 2.3	32.2 ± 6.7	136.4 ± 19.2	4.7 ± 0.5
2	Model	55.1 ± 3.9	39.2 ± 2.6	31.4 ± 5.8	120.3 ± 16.9	4.5 ± 1.0
3	Low dose	54.9 ± 4.7	41.9 ± 3.2	34.2 ± 7.3	115.3 ± 26.6	4.8 ± 0.6
4	Medium dose	53.0 ± 3.3	38.7 ± 2.4	31.5 ± 4.5	120.3 ± 20.5	4.4 ± 0.7
5	High dose	51.9 ± 1.2	40.3 ± 1.1	33.6 ± 4.4	119.1 ± 22.7	4.3 ± 0.6
		**Cr (μmol/L)**	**Glu (mmol/L)**	**TG (mmol/L)**	**TC (mmol/L)**	
1	Control	29.0 ± 6.2	6.6 ± 0.5	0.6 ± 0.3	1.3 ± 0.2	
2	Model	28.7 ± 5.4	6.0 ± 1.3	0.4 ± 0.3	1.7 ± 0.3	
3	Low dose	27.4 ± 7.5	6.8 ± 0.6	0.5 ± 0.2	1.8 ± 0.1	
4	Medium dose	26.3 ± 2.9	6.7 ± 0.8	0.6 ± 0.2	1.3 ± 0.3	
5	High dose	25.5 ± 3.8	5.6 ± 0.7	0.7 ± 0.4	1.6 ± 0.4	

### 3.6 Cytokine measurement

**Figure 3:**
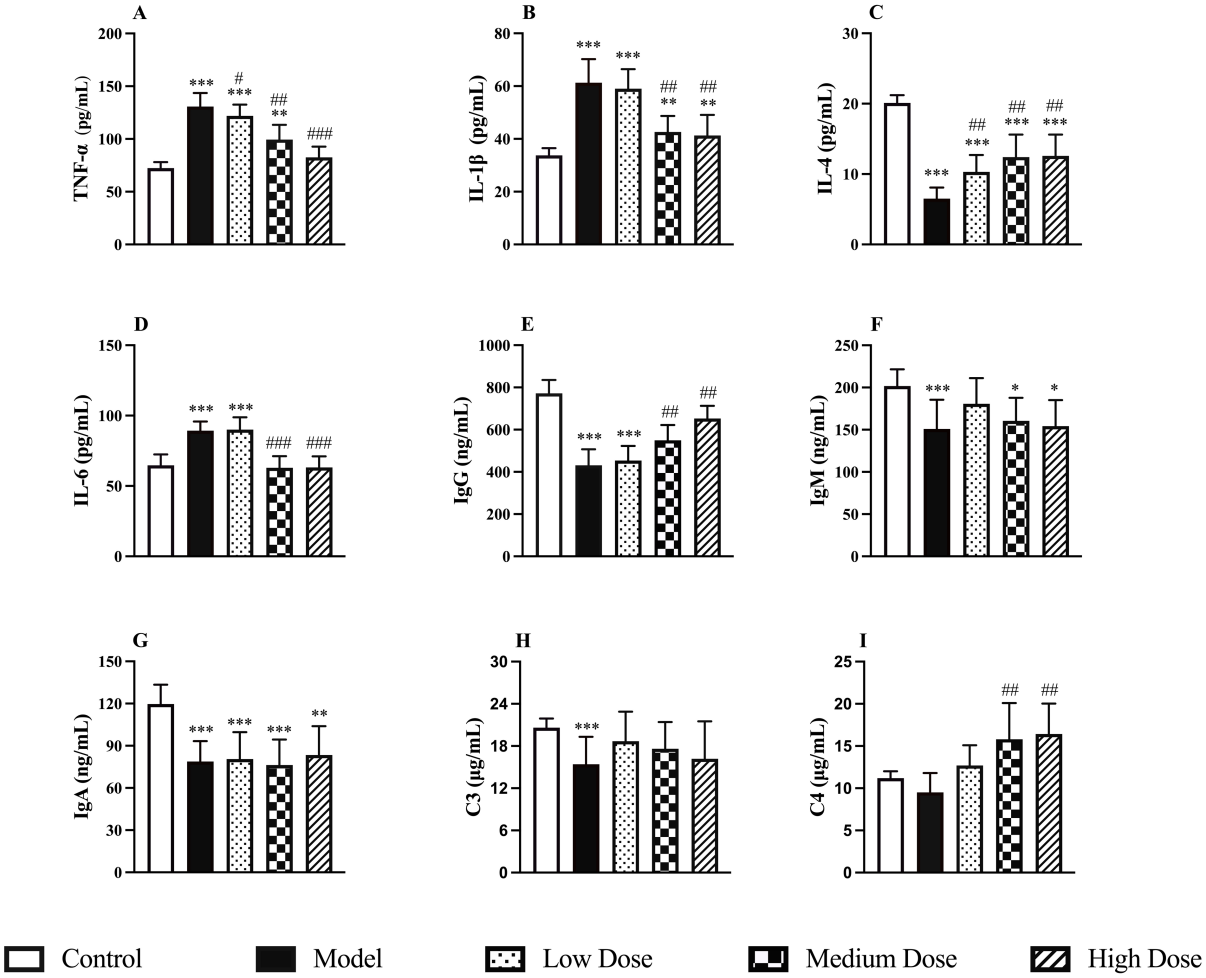
Effect of LEE on cytokine, immunoglobulin and complement levels in dexamethasone-induced immunosuppressed rats. Rats were intraperitoneally administrated dexamethasone (7.5 mg/kg, i.p., once daily for 7 consecutive days) to induce immunosuppression. Subsequently, animals in the treatment groups received daily oral doses of LEE for 21 consecutive days (low dose, 10 mg/kg; medium dose, 20 mg/kg; high dose, 40 mg/kg) The spleen was collected to assess the levels of cytokine (TNF-a, IL-1b, IL-4, and IL-6) **(A–D)**, immunoglobulins (IgG, IgM, and IgA) **(E–G)** and complement proteins (C3 and C4) **(H,I)** as described in the methods section. LEE significantly downregulated pro-inflammatory cytokines while restoring anti-inflammatory cytokine IL-4 and humoral immune factors (immunoglobulins and complements), indicating improved immune balance. Data are expressed as mean ± SD (*n* = 10). *,**,*** indicated statistically significant difference compared with the control group, and #,##,### indicated statistically significant difference compared with the model group (*p* < 0.05, *p* < 0.01, or *p* < 0.001).

Following dexamethasone administration, the model group exhibited significantly elevated levels of pro-inflammatory cytokines TNF-*α*, IL-1β, and IL-6, along with a marked reduction in the anti-inflammatory cytokine IL-4 compared to the control group (*p* < 0.001). Treatment with LEE significantly reversed these changes in a dose-dependent manner. In both the medium and high dose groups, TNF-α and IL-6 levels were restored to levels comparable to those of the control group (*p* > 0.05), while IL-1β levels were significantly reduced and IL-4 levels significantly increased (*p* < 0.01 or *p* < 0.001). These findings suggest that LEE can effectively regulate cytokine secretion and mitigate immunosuppression. Detailed results are presented in Figures 3A–D.

### 3.7 Immunoglobulin and complement measurement

Dexamethasone administration significantly reduced the levels of IgG, IgM, and IgA in the spleen compared to the control group (*p* < 0.001), indicating a marked immunosuppressive effect. Treatment with LEE notably reversed this trend. In particular, the medium and high dose groups showed significantly increased IgG levels compared to the model group (*p* < 0.01). In terms of complement proteins, C3 levels were significantly decreased following dexamethasone treatment (*p* < 0.001), whereas C4 levels remained unchanged (*p* > 0.05). After treatment with LEE, C3 levels increased, though not significantly compared to the control group (*p* > 0.05). However, C4 levels were significantly elevated in both the medium and high dose groups compared to the control group (*p* < 0.01), suggesting a possible regulatory role of the extract on complement pathways. These findings indicate that LEE can partially restore immunoglobulin levels and modulate complement activity, thereby contributing to the improvement of immune function under immunosuppressed conditions. The detailed results are presented in Figures 3E–I.

### 3.8 Real-time quantitative PCR expression

**Figure 4:**
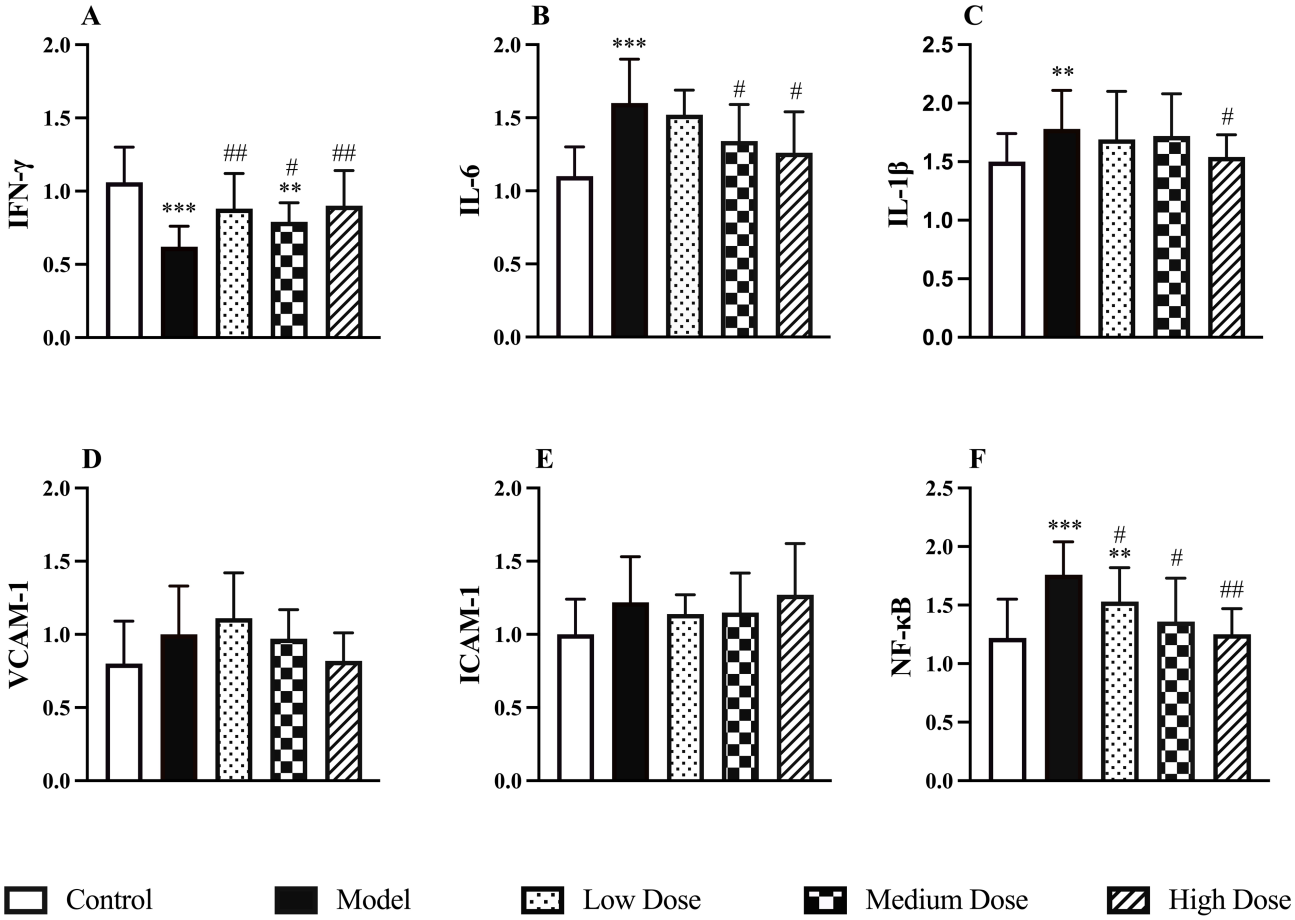
Effect of LEE on relative mRNA expressions levels in dexamethasone-induced immunosuppressed rats. Rats were intraperitoneally administrated dexamethasone (7.5 mg/kg, i.p., once daily for 7 consecutive days), followed by daily oral administration of LEE at different doses for 21 consecutive days (low dose, 10 mg/kg; medium dose, 20 mg/kg; high dose, 40 mg/kg). LEE treatment downregulated pro-inflammatory and adhesion-related genes (IL-6, IL-1β, VCAM-1, ICAM-1, NF-kB), while restoring IFN-γ expression, suggesting attenuation of inflammatory signaling and improved immune regulation. The spleen was harvested for quantitative PCR analysis of IFN-γ, IL-6, IL-1β, VCAM-1, ICAM-1, and NF-KB mRNA levels **(A–F)**. Data are expressed as mean ± SD (*n* = 10). *,**,*** indicated statistically significant difference compared with the control group, and #,##,### indicated statistically significant difference compared with the model group (*p* < 0.05, *p* < 0.01, or *p* < 0.001).

Compared to the control group, dexamethasone-induced immunosuppression significantly reduced the mRNA expression of IFN-*γ* in the spleen, while the mRNA levels of IL-6, IL-1β and NF-κB were significantly upregulated (*p* < 0.01 or *p* < 0.001). Following treatment with LEE, the medium and high dose groups exhibited a significant increase in IFN-γ expression and decreases in IL-6, IL-1β, and NF-κB expression (*p* < 0.05 or *p* < 0.01), indicating that the extract effectively regulated immune-related gene transcription. These findings are illustrated in Figure 4.

### 3.9 Lymphocyte subtype analysis

**Figure 5:**
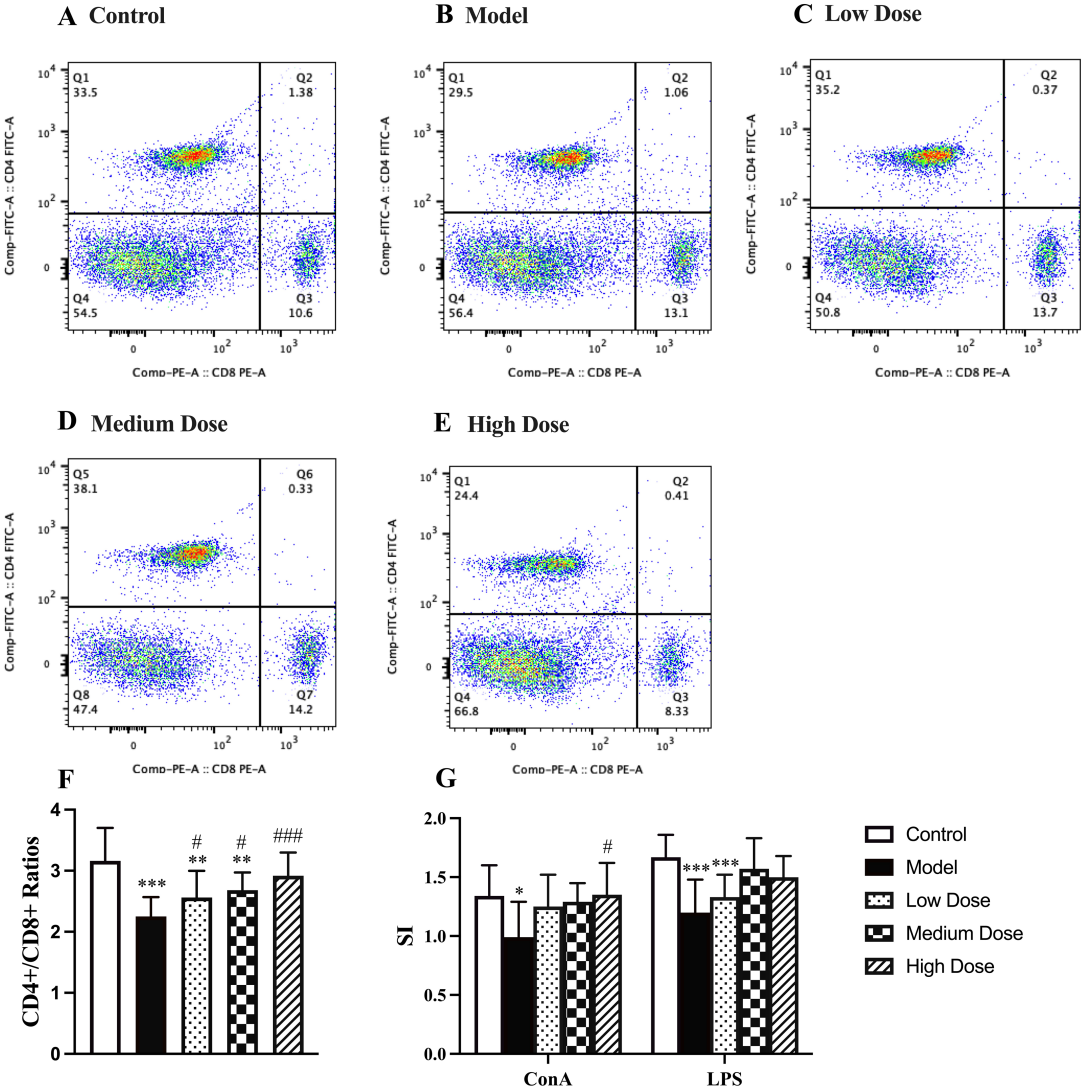
Effect of LEE on lymphocyte subtype ratio and lymphocyte proliferation stimulation index (SI) in dexamethasone-induced immunosuppressed rats. Rats were intraperitoneally administrated dexamethasone (7.5 mg/kg, i.p., once daily for 7 consecutive days) to induce immunosuppression, followed by daily oral administration of LEE at various doses for 21 consecutive days (low dose, 10 mg/kg; medium dose, 20 mg/kg; high dose, 40 mg/kg). At the end of the treatment period, spleens were harvested, and splenic lymphocytes were prepared. The CD4^+^/CD8^+^ T cell ratio was analyzed by flow cytometry **(A–E)**, as described in the methods section. For lymphocyte proliferation analysis, isolated splenic lymphocytes were cultured *in vitro* and stimulated with either ConA (T cell proliferation) or LPS (B cell proliferation). Proliferative responses were evaluated using the MTT assay, and the stimulation index (SI) was calculated accordingly. LEE significantly increased the CD4^+^/CD8^+^ ratio and enhanced both T and B cell proliferation, indicating restoration of cellular immune competence. Data are presented as mean ± standard deviation (SD) (*n* = 10). *,**,*** indicated statistically significant difference compared with the control group, and #,##,### indicated statistically significant difference compared with the model group (**F**, CD4^+^/CD8^+^ ratios) (**G**, SI) (*p* < 0.05, *p* < 0.01, or *p* < 0.001).

As shown in Figures 5A–F, dexamethasone administration significantly reduced the CD4^+^/CD8^+^ T lymphocyte ratio compared to the control group (*p* < 0.001), indicating an imbalance in T cell subpopulations due to immunosuppression. Treatment with LEE mitigated this effect in a dose-dependent manner. Notably, the high dose group restored the CD4^+^/CD8^+^ ratio to a level comparable to that of the control group (*p* > 0.05), suggesting that LEE can effectively regulate T lymphocyte homeostasis under immunosuppressive conditions.

### 3.10 Lymphocyte proliferation assay

As shown in Figure 5G, dexamethasone treatment significantly suppressed lymphocyte proliferation, as indicated by a decreased SI in response to both ConA and LPS. LEE treatment effectively alleviated this suppression. Specifically, the high dose group significantly restored the ConA-induced SI compared to the model group (*p* < 0.05), although no significant difference was observed compared to the control group (*p* > 0.05). For LPS-induced SI, both the medium and high dose groups showed significant improvement compared to the model group, and the values remained statistically indistinguishable from those of the control group (*p* > 0.05). These results suggest that LEE enhances both T cell (ConA-induced) and B cell (LPS-induced) proliferative responses under immunosuppressive conditions.

### 3.11 Organ weights

**Figure 6:**
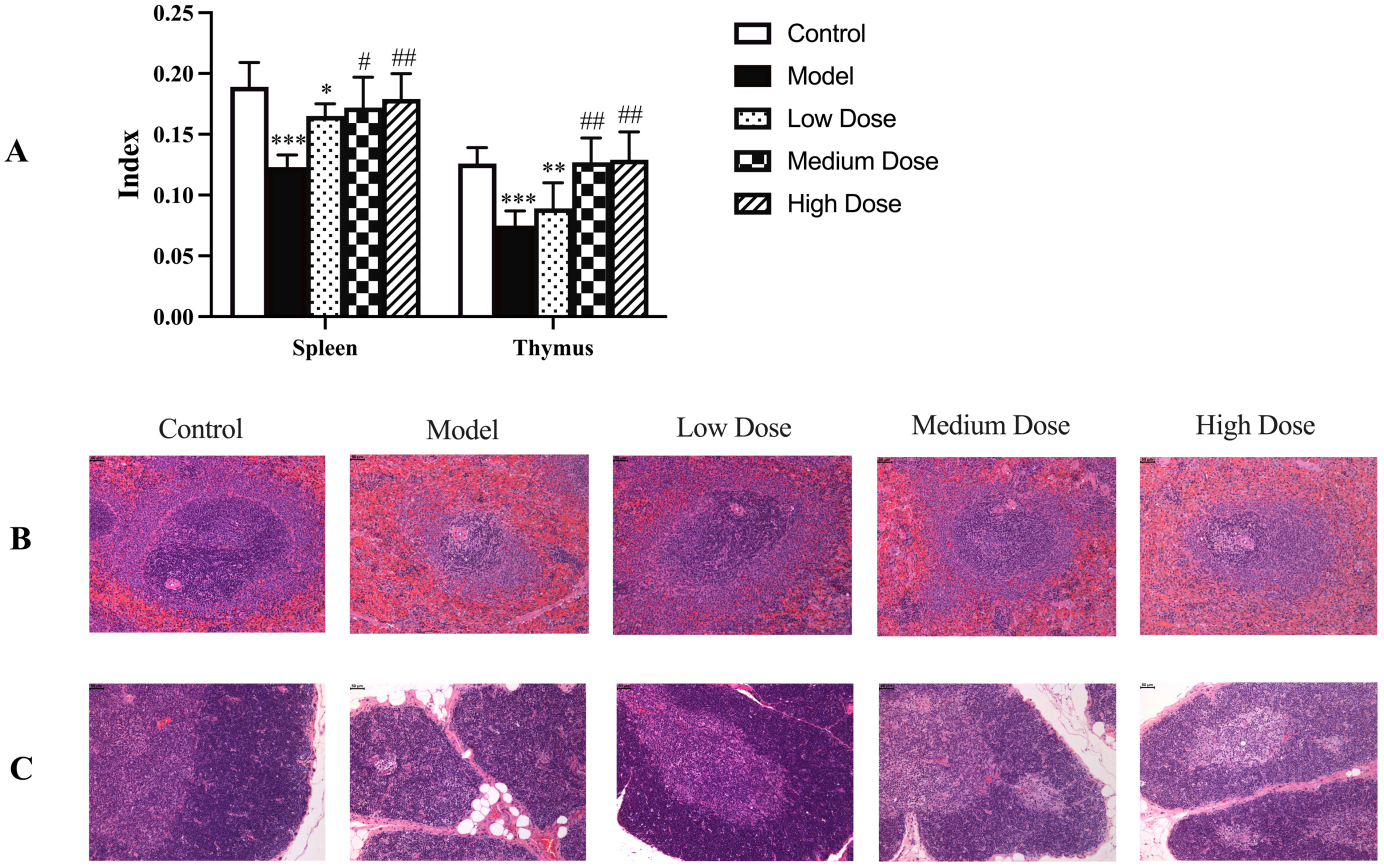
Effect of LEE on immune organ indices and histopathology changes in dexamethasone-induced immunosuppressed rats. Rats were intraperitoneally administrated dexamethasone (7.5 mg/kg, i.p., once daily for 7 consecutive days) to induce immunosuppression. Following this, animals in the LEE-treated groups received daily oral administration of LEE at various doses for 21 consecutive days (low dose, 10 mg/kg; medium dose, 20 mg/kg; high dose, 40 mg/kg). At the end of the treatment, the spleen and thymus were excised, and organ indices (organ-body weight ratio) were calculated as described in the methods section **(A)**. For histopathological evaluation, the collected organs were fixed, sectioned, and stained with hematoxylin and eosin (HE) according to standard protocols. Representative histological images of spleen and thymus tissues are presented **(B,C)** showed that LEE mitigated dexamethasone-induced atrophy and structural damage, with clearer tissue architecture compared to the model group. Scale bar = 50 μm. Quantitative data are expressed as mean ± standard deviation (SD) (*n* = 10). *,**,*** indicated statistically significant difference compared with the control group, and #,##,### indicated statistically significant difference compared with the model group (*p* < 0.05, *p* < 0.01, or *p* < 0.001).

As shown in Figure 6A, dexamethasone administration significantly reduced both spleen and thymus indices compared to the control group (*p* < 0.001), reflecting immunosuppression-induced atrophy of immune organs. Oral administration of LEE effectively ameliorated these reductions. Notably, the medium and high dose LEE groups exhibited significantly higher spleen and thymus indices than the model group (*p* < 0.05 or *p* < 0.01), with no statistically significant differences from the control group (*p* > 0.05). These findings indicate that LEE can restore immune organ indices and potentially reverse dexamethasone-induced immune organ atrophy.

### 3.12 Histopathology examination

As shown in Figures 6B,C, dexamethasone-induced immunosuppression caused marked histopathological alterations in the spleen and thymus, including reduced cortical-medullary differentiation, lymphocyte depletion, and tissue atrophy. These pathological changes are consistent with structural damage and functional decline of immune organs. In contrast, treatment with LEE notably alleviated these histological lesions. The medium and high dose groups exhibited relatively preserved tissue architecture, with improved lymphocyte density and restoration of thymic and splenic microstructures. These findings further support the protective effect of LEE against immunosuppression-induced organ damage.

### 3.13 Computational system pharmacology analysis

#### 3.13.1 Potential target genes and the PPI network analysis of LEE in immunosuppression protection

**Figure 7:**
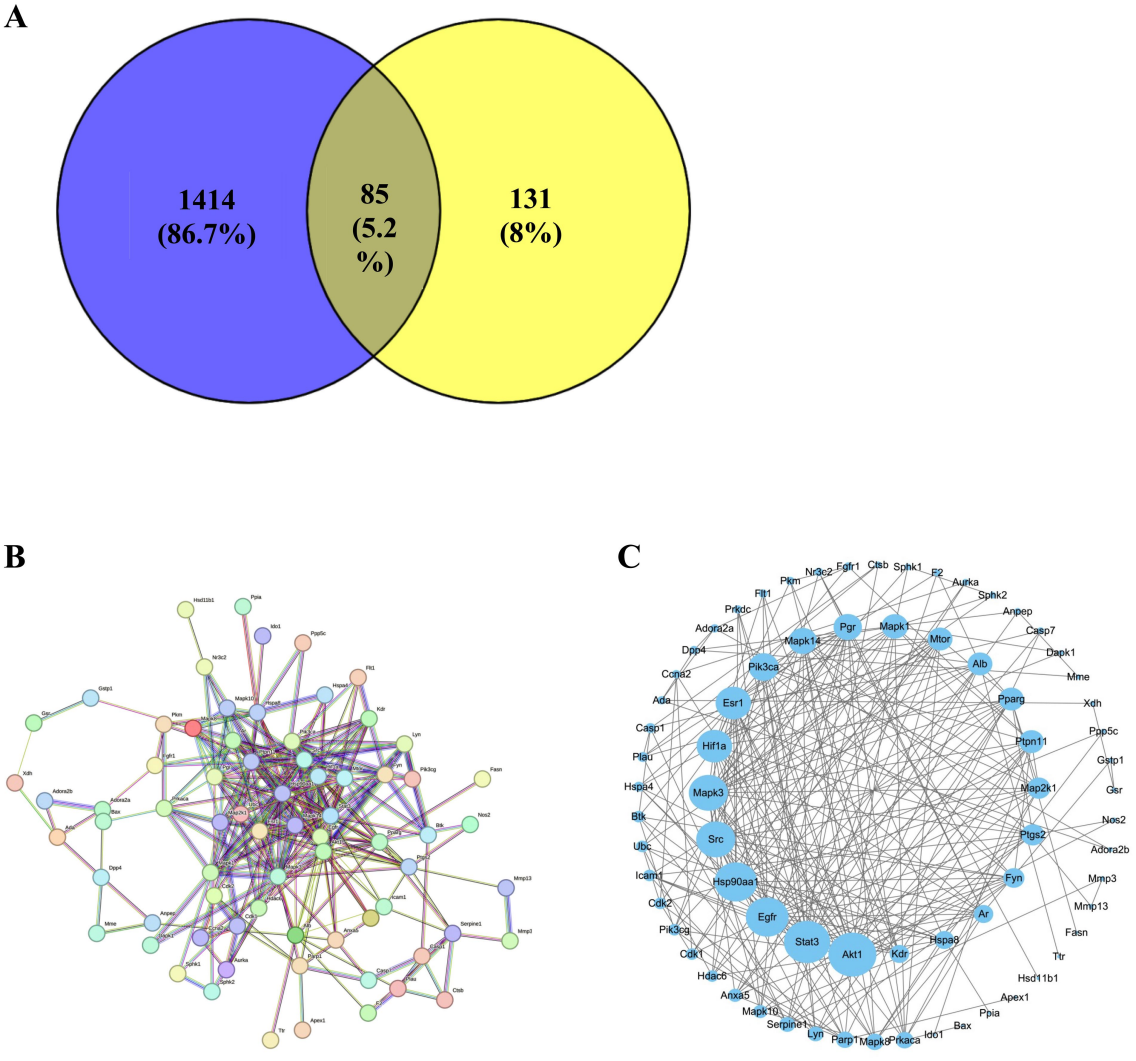
Potential target genes of LEE in immunosuppression. **(A)** The STRING protein–protein interaction (PPI) network of 85 potential target. **(B)** Venn diagram showing the intersection of differentially expressed genes in immunosuppression and predicted targets of LEE. **(C)** Visualized PPI network constructed using Cytoscape, displaying topological features of the target genes. These analyses suggested that LEE exerted immunomodulatory effects through a core set of overlapping genes, providing mechanistic insight into its potential molecular targets.

A total of 85 overlapping genes were identified by intersecting differentially expressed genes associated with immunosuppression and predicted targets of active compounds in LEE (Figure 7A). A protein–protein interaction (PPI) network was constructed using the STRING database, comprising 85 nodes and 276 edges (Figure 7B). This network was further visualized and analyzed using Cytoscape software (Figure 7C). Topological analysis of the PPI network indicated that Akt1, Mapk3, Pik3ca, Mapk14, and Mapk1 were core nodes with high connectivity (degree > 10), suggesting they may serve as key targets through which LEE exerts its immunomodulatory effects (see Supplementary Table 6).

#### 3.13.2 Potential action mechanisms of LEE in immunosuppression protection

**Figure 8:**
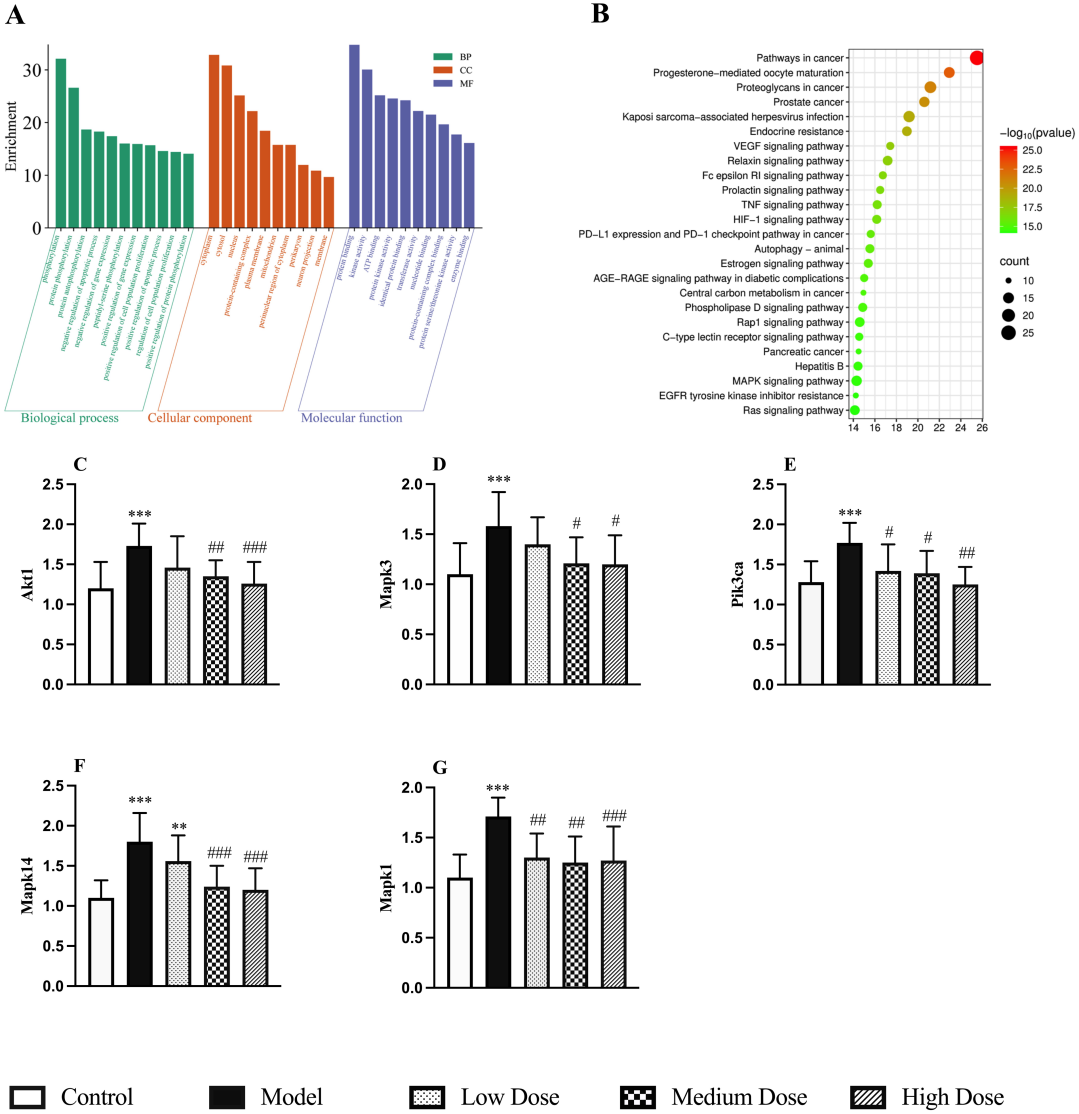
Potential action mechanisms of LEE and validation of potential target genes of LEE in immunosuppression. **(A)** GO enrichment analysis of biological processes, molecular functions, and cellular components associated with LEE target genes. **(B)** KEGG pathway enrichment analysis highlighting the MAPK signaling pathway as the most relevant mechanism. **(C–G)** RT-qPCR validation of mRNA expression levels of five key target genes (Akt1, Mapk3, Pik3ca, Mapk14, and Mapkl) in spleen tissues of rats treated with LEE. These results indicated that LEE regulated immune responses mainly through the MAPK signaling axis and modulated expression of critical genes involved in inflammatory and immune pathways, thereby supporting the network pharmacology predictions. Data are expressed as mean ± SD (n = 10). *,**,*** indicated statistically significant difference compared with the control group, and #,##,### indicated statistically significant difference compared with the model group (*p* < 0.05, *p* < 0.01, or *p* < 0.001).

The 85 potential target genes were subjected to GO and KEGG enrichment analysis using the Metascape platform. GO enrichment results suggested that LEE may exert its therapeutic effects against inflammation-induced immunosuppression by modulating key biological processes such as phosphorylation, protein binding, gene expression, apoptosis, and cell proliferation. These effects are associated with cellular components including the cytoplasm, cytosol, nucleus, protein-containing complexes, and the plasma membrane (Figure 8A). KEGG pathway analysis revealed that the MAPK signaling pathway showed the highest level of enrichment among the those potentially implicated in the immunoregulatory effects of LEE (Figure 8B). This pathway, along with others such as PI3K-Akt, is well known for its involvement in modulating immune activation, promoting cell survival, and maintaining inflammatory homeostasis. To validate the relevance of these pathways, five representative hub genes were selected for RT-qPCR verification (Akt1, Mapk3, Pik3ca, Mapk14, and Mapk1), all of which are central to the aforementioned signaling cascades. The RT-qPCR results showed that LEE treatment significantly upregulated the mRNA expression levels of these genes in rat spleen tissue (*p* < 0.05, *p* < 0.01, or *p* < 0.001), providing experimental confirmation of the bioinformatics predictions and supporting the immunorestorative role of LEE under dexamethasone-induced immunosuppression (Figures 8C–G).

## 4 Discussion

The present study demonstrated that *Lithospermum erythrorhizon* extract (LEE) significantly mitigated dexamethasone-induced immunosuppression in rats by modulating multiple immune parameters, restoring immune organ indices, regulating cytokine profiles, and altering the expression of key immune-related genes. These findings highlight the potential of LEE as a promising immunopotentiator with anti-inflammatory and immunoregulatory properties.

Immunosuppression poses significant health risks and can lead to more severe diseases. The body is usually exposed to many harmful factors such as inflammation, chronic diseases, chemotherapy and radiation therapy, autoimmune disorders or aging which may aggravate immunosuppression. These changes underscore the severe consequences of immunosuppression on overall health. Heikki et al. has documented these effects, highlighting the correlation between immunosuppression, weight loss, and hematological alterations (28). Studies have shown that dexamethasone promoted the secretion of pro-inflammatory cytokines while inhibiting the production of immunoglobulins and complement, ultimately impairing the function of immune organs (29–32). Given the established link between chronic inflammation and immunosuppressive states, we selected dexamethasone as a model compound to mimic chronic inflammation-induced immunosuppression, allowing for the evaluation of potential therapeutic interventions. In the present study, dexamethasone could decrease the body weights (Supplementary Table 4), hematology parameters of WBC and LYMPH% (Table 3), IL-4 (Figure 3C), IgG, IgM, IgA, C3, and C4 (Figures 3E–I), CD4+/CD8 + ratio (Figure 5F), lymphocyte proliferation SI (Figure 5G), the spleen and thymus-body ratios (Figure 6A), increase the TNF-*α*, IL-1β, and IL-6 (Figures 3A,B,D), and cause the change of immune gene mRNA expression (Figure 4), resulting in significantly spleen and thymus atrophy (Figures 6A,B).

Immunosuppression is often accompanied by a deterioration in general health status, typically manifesting as body weight loss and reduced appetite due to metabolic imbalances and inflammatory stress (33). In this study, rats treated with dexamethasone exhibited a significant reduction in body weight and food consumption, consistent with previously reported models of glucocorticoid-induced immunosuppression (34). Oral administration of LEE significantly mitigated these effects, suggesting an overall improvement in metabolic and systemic health (Supplementary Tables 4, 5). The observed improvements reflect LEE’s capacity to counteract inflammation-induced catabolic effects and support physiological homeostasis. Hematological indicators such as WBC and LYMPH% are key parameters in evaluating immune competence. Dexamethasone treatment significantly suppressed these indices, reflecting leukopenia and lymphopenia, both of which are hallmarks of immunosuppression (35). Our results showed that LEE administration significantly reversed these declines, indicating enhanced hematopoietic and immunoregulatory function (Table 3). Additionally, serum biochemical indicators remained largely stable across all LEE-treated groups (Table 4), suggesting that LEE does not induce hepatic or renal toxicity at the administered doses, which is consistent with its traditional use in herbal medicine (36). Although certain naphthoquinone derivatives from *Lithospermum erythrorhizon*, particularly shikonin, have been reported to exhibit redox-active properties that could theoretically provoke oxidative stress or off-target cytotoxicity at high concentrations (37), such effects were not evident in this study. Neither weight loss nor biochemical signs of oxidative injury were observed. These findings suggest that, under the dosing regimen used here, LEE exhibits a favorable safety profile without evidence of pro-oxidative liabilities. Nonetheless, further mechanistic and toxicokinetic studies would be warranted to fully characterize long-term safety and potential tissue-specific effects of shikonin-containing extracts.

Chronic inflammation disrupts cytokine equilibrium and impairs both cellular and humoral immunity (7). Consistent with this, dexamethasone elevated pro-inflammatory cytokines (TNF-*α*, IL-1β, IL-6) while reducing IL-4. LEE treatment effectively downregulated the expression of pro-inflammatory cytokines while restoring anti-inflammatory cytokines (Figures 3A–D), suggesting a rebalancing of Th1/Th2 responses. Moreover, LEE significantly elevated serum levels of immunoglobulins (IgG, IgM, IgA) and complements (C3, C4) (Figures 3E–I), indicating restoration of humoral immune competence. These findings align with previous reports on shikonin’s immunomodulatory effects (38, 39).

RT-qPCR analysis further supported the immunoregulatory role of LEE by showing restored mRNA expression of IFN-*γ* and suppressed expression of IL-6, IL-1β, NF-κB, VCAM-1, and ICAM-1 (Figure 4). NF-κB, a master regulator of inflammation, plays a pivotal role in immune dysfunction under chronic stress (40). The suppression of NF-κB and its downstream adhesion molecules by LEE suggests a molecular mechanism through which it may inhibit leukocyte migration and cytokine storms, thereby alleviating systemic inflammation. Immunosuppression typically alters lymphocyte subtypes, especially the CD4+/CD8 + ratio, leading to impaired adaptive immunity (41). Our results showed that LEE restored the CD4+/CD8 + balance and enhanced ConA- and LPS-induced lymphocyte proliferation (Figure 5). These outcomes confirm that LEE supports both helper T cell function and B cell activation. This is consistent with studies reporting that shikonin derivatives can promote dendritic cell maturation and antigen presentation (42, 43).

The spleen and thymus are primary lymphoid organs essential for immune cell development and homeostasis (44). In this study, dexamethasone administration induced notable atrophy of these organs, as reflected by the significant decline in organ-body weight ratios and histopathological evidence of structural disintegration. HE revealed reduced white pulp density in the spleen and cortical thinning in the thymus, both indicative of immunosuppressive damage (Figures 6B,C). Remarkably, LEE treatment reversed these pathological changes in a dose-dependent manner, significantly restoring spleen and thymus indices and preserving tissue architecture (Figure 6A). These findings suggest that LEE confers protective effects on central immune organs, thereby enhancing host immune competence under conditions of pharmacologically induced immunosuppression.

To further explore the mechanisms underlying LEE’s immunoregulatory and anti-inflammatory effects, we employed a network pharmacology approach. From the intersection of predicted LEE-related targets and immunosuppression-associated genes, 85 overlapping targets were identified. Protein–protein interaction (PPI) analysis revealed several key hub genes, including Akt1, Mapk3, Pik3ca, Mapk14, and Mapk1—all of which are central regulators in immune and inflammatory signaling pathways (Figure 7; Supplementary Table 6). KEGG pathway enrichment analysis identified the MAPK signaling cascade as the most significantly involved pathway (Figure 8), implicating its role in the modulation of cytokine production and immune cell activity (45). To validate these findings, RT-qPCR analysis demonstrated that LEE treatment significantly upregulated the mRNA expression of these five hub genes in spleen tissue (Figure 8), confirming their involvement in the observed immunomodulatory and anti-inflammatory effects. Notably, the MAPK pathway, particularly the p38 and JNK branches, has been well-documented to regulate pro-inflammatory cytokine expression (e.g., TNF-*α*, IL-1β) and T cell differentiation (46). These results collectively suggest that LEE mitigates immunosuppression induced by inflammatory, at least in part, by modulating MAPK-dependent inflammatory and immune signaling pathways. While several natural compounds—such as curcumin, resveratrol—have been investigated for their immunomodulatory potential (47, 48), LEE appears to exert a more selective mechanisms and broader spectrum of regulatory activity both inflammatory and structural components of the immune microenvironment. Unlike many conventional immunopotentiators, which may enhance immune activation at the expense of inflammatory control, LEE appears to rebalance immune homeostasis by concurrently restoring adaptive immunity and downregulating excessive inflammatory signals. These properties, combined with its favorable safety profile, position LEE as a distinctive botanical candidate with dual immunorestorative and anti-inflammatory capabilities, meriting further investigation alongside established immunomodulatory agents. Taken together, our findings demonstrate that LEE exerts multifaceted immunoprotective effects by preserving immune organ structure, enhancing key immune parameters, and regulating molecular pathways associated with inflammation and immune activation. Such balanced regulation is particularly relevant in veterinary contexts where overactivation of the immune system may predispose to autoimmune complications or chronic inflammatory diseases. Thus, LEE may represent a promising candidate for integrative veterinary approaches aimed at improving animal health, reducing reliance on antibiotics, and enhancing resilience against infections. These results support the therapeutic potential of LEE as a natural immunopotentiator for the management of inflammation-related immune dysregulation. Given its dual regulatory activity and favorable safety profile, LEE holds promise as a translational candidate for future clinical development targeting immune dysfunction and chronic inflammatory conditions.

While this study demonstrated that LEE mitigates dexamethasone-induced immunosuppression in rats, further work is warranted. Validation in diverse preclinical models, including large animals, is needed to confirm reproducibility and assess species-specific responses. Pharmacokinetic and toxicokinetic studies should define optimal dosing and long-term safety. Ultimately, translational studies—such as pilot human trials or controlled veterinary trials—will be essential to establish the therapeutic value of LEE in managing immunosuppression and chronic inflammation.

## 5 Conclusion

In conclusion, this study demonstrates that LEE exerts potent immunomodulatory effects in a rat model of dexamethasone-induced immunosuppression. LEE significantly improved body weight, hematological parameters, cytokine balance, immunoglobulin and complement levels, lymphocyte proliferation, and immune organ indices, while alleviating thymus and spleen atrophy. Mechanistically, LEE modulated the expression of key immune-related genes such as IFN-*γ*, IL-1β, and NF-κB, and was found to target multiple signaling pathways through network pharmacology analysis, particularly the MAPK pathway. RT-qPCR validation further confirmed the upregulation of central nodes including Akt1, Mapk3, Mapk14, and Pik3ca, suggesting that LEE may restore immune homeostasis via the p38/MAPK signaling axis. These findings provide pharmacological evidence supporting the potential application of LEE as a natural immunopotentiator for preventing or alleviating inflammation-associated immunosuppression. Future studies are warranted to explore its clinical relevance and to further elucidate its molecular targets in disease contexts.

## Data Availability

The datasets presented in this study can be found in online repositories. The names of the repository/repositories and accession number(s) can be found in the article/Supplementary material.

## Footnotes

^1^ https://tcmsp-e.com/

^2^ http://www.swisstargetprediction.ch/

^3^ http://sea.bkslab.org/

^4^ https://www.uniprot.org/

^5^ https://bioinfogp.cnb.csic.es/tools/venny/

^6^ https://string-db.org/

^7^ https://metascape.org/

## Glossary

Akt1: Protein Kinase B*α*
Alb: Albumin
ALT: Alanine aminotransferase
AST: Aspartate Aminotransferase
BUN: Urea Nitrogen
C3: Complement 3
C4: Complement 4
ConA: Concanavalin A
Cr: Creatinine
EDTA-K2: EDTA Dipotassium Salt
Glu: Glucose
GO: Gene Ontology
HE: Hematoxylin and Eosin
HPLC: High Performance Liquid Chromatography
ICAM-1: Intercellular Adhesion Molecule 1
IFN-γ: Interferon-γ
IgA: Immunoglobulin A
IgG: Immunoglobulin G
IgM: Immunoglobulin M
IL-1β: Interleukin-1β
IL-2: Interleukin-2
IL-4: Interleukin-4
IL-6: Interleukin-6
JNK: c-Jun N-terminal Kinase
KEGG: Kyoto Encyclopedia of Genes and Genomes
LEE: *Lithospermum Erythrorhizon* Extract
LPS: Lipopolysaccharide
LYMPH%: Percentage of Lymphocytes
MAPK: Mitogen-activated Protein Kinase (p38)
MAP K1: Mitogen-activated Protein Kinase 1 (ERK2)
MAP K3: Mitogen-activated Protein Kinase 3 (ERK1)
MAP K14: Mitogen-activated Protein Kinase 14 (p38α)
mRNA: Messenger Ribonucleic Acid
NF-κB: Nuclear Factor κB
OD: Optical Density
PPI: Protein–protein Interaction
RT-qPCR: Real-time Quantitative PCR
SEA: Similarity Ensemble Approach
SI: Stimulation Index
STRING: Search Tool for the Retrieval of Interacting Genes/Proteins
TC: Total Cholesterol
TCMSP: Traditional Chinese Medicine Systems Pharmacology
TG: Triglyceride
TNF-α: Tumor Necrosis Factor-α
TP: Total Protein
VCAM-1: Vascular Cell Adhesion Molecule 1
WBC: White Blood Cell
