# Supplementary material for: Lithospermum erythrorhizon extract alleviates immunosuppression via MAPK signaling pathway
Source: Front Vet Sci. 2025 Sep 29;12:1654212. doi: 10.3389/fvets.2025.1654212 (PMC12516705; doi:10.3389/fvets.2025.1654212)
Supplement: Supplementary file 1 [file Table_1.DOCX]

**SUPPLEMENTARY** **TABLES**

STable 1 Hematology Parameters

| Parameters | Abbreviation | Unit | Method |
| --- | --- | --- | --- |
| White Blood Count | WBC | 10^9^/L | Electrical Impedance Method and Optical Method |
| Neutrophils | NEUT | % | Flow Cytometry for Semiconductor Lasers |
| Lymphocytes | LYMPH | % | Flow Cytometry for Semiconductor Lasers |
| Monocytes | MONO | % | Flow Cytometry for Semiconductor Lasers |
| Eosinophils | EO | % | Flow Cytometry for Semiconductor Lasers |
| Basophil Cells | BASO | % | Flow Cytometry for Semiconductor Lasers |
| Red Blood Count | RBC | 10^12^/L | Electrical Impedance Method and Optical Method |
| Hemoglobin | Hb | g/dL | SLS-Hemoglobin Method |
| Hematocrit | Hct | % | Calculation: (MCV x RBC)/10 |
| Mean Corpuscular Volume | MCV | fL | Electrical Impedance Method and Optical Method |
| Mean Corpuscular Hemoglobin | MCH | pg | Calculation: Hb/RBC x 10^3^ |
| Mean Corpuscular Hemoglobin Concentration | MCHC | g/dL | Calculation: Hb/(MCV x RBC) x 10^6^ |

STable 2 Serum Chemistry Parameters

| Parameters | Abbreviation | Unit | Method |
| --- | --- | --- | --- |
| Aspartate Aminotransferase | AST | U/L | Colorimetry |
| Alanine Aminotransferase | ALT | U/L | IFCC |
| Total Protein | TP | g/L | Colorimetry |
| Albumin | Alb | g/L | Colorimetry |
| Glucose | Glu | mmol/L | Hexokinase Method |
| Urea Nitrogen | BUN | mmol/L | Kinetic Method of Urease and Glutamate Dehydrogenase |
| Creatinine | Cr | µmol/L | Picric Acid |
| Total Cholesterol | TC | mmol/L | Enzyme Colorimetry |
| Triglyceride | TG | mmol/L | End Point Method |

STable 3 Sequences used for Real-time qPCR Assay

| Gene | Primer Sequence (5'-3') | Reverse Sequence (5'-3') | Size (bp) |
| --- | --- | --- | --- |
| IFN-γ | CATTCAGATGTAGCGGATAA | TTTCGCTTCCCTGTTTTA | 281 |
| IL-6 | AGCCACTCACCTCTTCA | TCTTTGCTGCTTTCACA | 115 |
| IL-1β | CGTGCAATGATGACTTTGTCTGT | AGAGCCTTCAGCATGTGTGG | 167 |
| VCAM-1 | CTGGGAAGCTGGAACGAAGT | GCCAAACACTTGACCGTGAC | 115 |
| ICAM-1 | CTGGGCTTGGAGACTCAGTG | CCACACTCTCCGGAAACGAA | 175 |
| NF-κB | TGACGGGAGGGGAAGAAATC | TGAACAAACACGGAAGCTGG | 91 |
| β-actin | TGTCACCAACTGGGACGATA | GGGGTGTTGAAGGTCTCAAA | 138 |
| Akt1 | ATGAACGACGTAGCCATTGTG | TTGTAGCCAATAAAGGTGCCAT | 104 |
| Mapk3 | TCCGCCATGAGAATGTTATAGGC | GGTGGTGTTGATAAGCAGATTGG | 150 |
| Pik3ca | CCACGACCATCTTCGGGTG | GGGGAGTAAACATTCCACTAGGA | 147 |
| Mapk14 | TGACCCTTATGACCAGTCCTTT | GTCAGGCTCTTCCACTCATCTAT | 150 |
| Mapk1 | GCACCAACCATCGAGCAAAT | CTTGAGGTCACGGTGCAGAA | 179 |
| GAPDH | GTCAAGGCTGAGAACGGGAA | AAATGAGCCCCAGCCTTCTC | 158 |

STable 4 Body Weights (Mean±S.D., n=20)

| SN | Group | Pre- Dexamethasone | Day 8 | Day 15 | Day 22 | Day 29 |
| --- | --- | --- | --- | --- | --- | --- |
| 1 | Control | 100.2±6.4 | 112.4±7.3^a^ | 125.7±10.1^a^ | 140.1±8.5^a^ | 183.6±8.8^a^ |
| 2 | Model | 105.3±4.2 | 100.7±5.4^c^ | 108.4±9.3^c^ | 115.2±7.7^c^ | 144.1±9.4^c^ |
| 3 | Low Dose | 98.5±8.1 | 102.2±7.8^bc^ | 117.9±8.2^abc^ | 130.0±6.9^b^ | 160.7±8.2^c^ |
| 4 | Medium Dose | 92.9±10.6 | 109.8±4.9^b^ | 123.4±9.2^ab^ | 138.5±10.2^a^ | 190.6±12.7^a^ |
| 5 | High Dose | 103.0±5.8 | 110.1±6.1^b^ | 122.5±7.8^ab^ | 132.9±8.4^ab^ | 173.7±15.2^ab^ |

Notes: Different letters are significantly different (*P* < 0.05, *P* < 0.01or *P* < 0.001).

STable 5 Food Consumption (Mean±S.D., n=20)

| SN | Group | Days after Administration | | | |
| --- | --- | --- | --- | --- | --- |
|  |  | D1-D8 | D8-D15 | D15-D22 | D22-D29  D29-D30 |
| 1 | Control | 22±0 | 23±0 | 27±0 | 27±0 |
| 2 | Model | 21±0 | 22±2 | 26±0 | 25±1 |
| 3 | Low Dose | 15±4 | 25±1 | 28±0 | 27±0 |
| 4 | Medium Dose | 14±0 | 26±5 | 32±1 | 37±9 |
| 5 | High Dose | 16±0 | 19±1 | 24±2 | 30±2 |

STable 6 Potential Target Genes of LEE in Immunosuppression Protection

| SN | Target | Degree |
| --- | --- | --- |
| 1 | Akt1 | 26 |
| 2 | Stat3 | 25 |
| 3 | Hsp90aa1 | 23 |
| 4 | Egfr | 23 |
| 5 | Mapk3 | 21 |
| 6 | Src | 21 |
| 7 | Esr1 | 19 |
| 8 | Hif1a | 19 |
| 9 | Pik3ca | 16 |
| 10 | Mapk14 | 15 |
| 11 | Pgr | 15 |
| 12 | Mapk1 | 14 |
| 13 | Pparg | 13 |
| 14 | Ptpn11 | 13 |
| 15 | Alb | 13 |
| 16 | Mtor | 13 |
| 17 | Ptgs2 | 12 |
| 18 | Map2k1 | 12 |
| 19 | Fyn | 11 |
| 20 | Hspa8 | 10 |
| 21 | Ar | 10 |
| 22 | Kdr | 10 |
| 23 | Parp1 | 9 |
| 24 | Prkaca | 9 |
| 25 | Mapk8 | 9 |
| 26 | Lyn | 8 |
| 27 | Serpine1 | 8 |
| 28 | Anxa5 | 7 |
| 29 | Mapk10 | 7 |
| 30 | Icam1 | 6 |
| 31 | Hdac6 | 6 |
| 32 | Pik3cg | 6 |
| 33 | Cdk1 | 6 |
| 34 | Btk | 6 |
| 35 | Cdk2 | 6 |
